# High‐Risk Screening for Late‐Onset Pompe Disease in Northern China: Prevalence, Genotypic Spectrum, and Diagnostic Challenges

**DOI:** 10.1002/jmd2.70138

**Published:** 2026-11-13

**Authors:** Hang Su, Duoling Li, Meng Yu, Junhong Guo, Xuefan Yu, Xiaojing Wei, Xueqin Song, Zhe Zhao, Mingming Ma, Kai Ma, Ting Chang, Xiangqi Cao, Xia Li, Juan Kang, Zaiqiang Zhang, Hong Han, Zhuyan Wang, Li Di, Ting Xiong, Sha Sun, Jun Wu, Shu Zhang, Cuiping Zhao, Yuan Sun, Weili Zhao, Zhiyun Wang, Yali Zhang, Haiping Wang, Ying Li, Chuanzhu Yan, Zhaoxia Wang, Yuying Zhao

**Affiliations:** ^1^ Department of Neurology, Shandong Key Laboratory of Mitochondrial Medicine and Rare Diseases, Research Institute of Neuromuscular and Neurodegenerative Diseases Qilu Hospital of Shandong University Jinan China; ^2^ Department of Neurology Peking University First Hospital Beijing China; ^3^ Department of Neurology The First Hospital of Shanxi Medical University Taiyuan China; ^4^ Department of Neurology and Neuroscience Center The First Affiliated Hospital of Jilin University Changchun Jilin China; ^5^ Department of Neurology The Second Hospital of Hebei Medical University and Neurological Laboratory of Hebei Province Shijiazhuang Hebei China; ^6^ Department of Neurology Hebei Medical University Third Hospital Shijiazhuang Hebei China; ^7^ Department of Neurology Affiliated People's Hospital of Zhengzhou University (Henan Provincial People's Hospital) Zhengzhou China; ^8^ Department of Neurology Jinan Children's Hospital (Children's Hospital Affiliated With Shandong University) Jinan China; ^9^ Department of Neurology, Tangdu Hospital The Fourth Military Medical University Xi'an China; ^10^ Department of Pediatric Neurology Xi'an Children's Hospital Xi'an China; ^11^ The Key Laboratory of Biomedical Information Engineering of Ministry of Education, School of Life Science and Technology, Institute of Health and Rehabilitation Science Xi'an Jiaotong University Xi'an China; ^12^ Department of Neurology Xi'an People's Hospital (Xi'an Fourth Hospital) Xi'an China; ^13^ Department of Neurology, China National Clinical Research Center for Neurological Diseases, Beijing Tiantan Hospital Capital Medical University Beijing China; ^14^ Department of Neurology Children's Hospital of Shanxi Taiyuan China; ^15^ Department of Neurology, Xuanwu Hospital Capital Medical University Beijing China; ^16^ Department of Neurology Xi'an Gaoxin Hospital Xi'an China; ^17^ Department of Neurology The First Affiliated Hospital of Zhengzhou University Zhengzhou China; ^18^ Department of Neurology The First Medical Center of Chinese PLA General Hospital Beijing China; ^19^ Department of Neurology, Qilu Hospital, Cheeloo College of Medicine Shandong University Qingdao China; ^20^ Department of Neurology Affiliated Hospital of Chifeng University Chifeng China; ^21^ Department of Neurology Tianjin First Central Hospital Tianjin China; ^22^ Department of Neurology Chifeng Municipal Hospital Chifeng China; ^23^ Department of Neurology The Affiliated Hospital of Qingdao University Qingdao China; ^24^ Medical Department of Rare Disease and Rare Blood Disorders Sanofi Shanghai China

**Keywords:** dried blood spot, *GAA*, high‐risk screening, late‐onset Pompe disease, Northern China

## Abstract

Late‐onset Pompe disease (LOPD), also known as late‐onset glycogen storage disease Type II, is caused by a genetic deficiency of the lysosomal enzyme acid α‐glucosidase (GAA). LOPD typically results in progressive weakness of the limb‐girdle and respiratory muscles and is often accompanied by the involvement of other systems, such as skeleton, smooth muscle, and the cerebrovascular system. The incidence of LOPD in China remains unknown, underscoring the need for large‐scale epidemiological studies to better characterize the clinical spectrum. This prospective, multicenter, observational cohort study was conducted across 39 neurology centers in 12 northern Chinese provinces. A novel pragmatic approach was utilized, which included the development and application of a standardized high‐risk profile for LOPD suspicion, derived from collective diagnostic and therapeutic experience throughout the region. Individuals meeting 2 or more criteria in this predefined high‐risk profile were enrolled. Subsequent screening involved initial analysis of GAA enzyme activity using dried blood spot (DBS) testing and patients with deficient enzyme activity underwent confirmatory genetic testing to establish a definitive LOPD diagnosis. Between January 2022 and December 2024, 1878 high‐risk patients were enrolled. Screening identified 46 patients (2.45%) diagnosed with LOPD based on DBS analysis and subsequent genetic confirmation. This study demonstrates a significant prevalence of LOPD among a clinically high‐risk population in Northern China. These findings underscore the critical importance of targeted screening programs for early identification. Early diagnosis enables timely initiation of enzyme replacement therapy (ERT), which can significantly mitigate the disease course, improve quality of life, and reduce long‐term healthcare burden.

## Synopsis

Targeted screening programs are critical for identifying individuals with LOPD; systematically screening suspected patients using DBS analysis for GAA enzyme deficiency and confirmatory genetic testing enables earlier diagnosis and therapeutic intervention.

## 1 Introduction

Pompe disease (PD) is a rare, autosomal recessive, progressive metabolic myopathy caused by pathogenic variants of the *GAA* gene, which encodes the lysosomal enzyme acid α‐glucosidase (GAA). These variants result in absent or significantly reduced GAA enzyme activity, leading to abnormal glycogen accumulation within lysosomes of cells in skeletal, cardiac, and smooth muscle. This accumulation impairs organ function, clinically manifesting as limb weakness, respiratory failure, and cardiac insufficiency [1, 2]. According to a 2024 meta‐analysis, the global birth prevalence of PD is approximately 1 in 50 000 live births [3], though the actual prevalence may be higher with the increasing implementation of newborn screening programs [4]. The predicted incidence in China is 1 in 40 000 [5].

PD is classified into infantile‐onset Pompe disease (IOPD) or late‐onset Pompe disease (LOPD). IOPD typically presents within the first year of life, characterized by severe GAA deficiency and primarily affecting skeletal and cardiac muscle. LOPD can manifest during childhood, adolescence, or adulthood, typically presenting insidiously, with progressive weakness of proximal limb‐girdle and axial muscles [1]. Early diagnosis is critical, as enzyme replacement therapy (ERT) can improve survival and motor function. However, in China, diagnostic delays average 7.2 years [6], often due to nonspecific symptoms (such as elevated creatine kinase [CK] and gastrointestinal, cerebrovascular, and spinal involvement) and misdiagnosis [1, 6, 7, 8, 9]. To improve detection, individuals meeting at least two of nine predefined clinical criteria were considered high‐risk, capturing those with nonspecific but converging features who might otherwise be overlooked.

High‐risk screening via dried blood spot (DBS) GAA activity testing, followed by genetic confirmation, has proven effective for identifying presymptomatic or early‐stage LOPD [10]. However, in real‐world studies, approximately 90% of patients with PD were from Europe and North America, while only 10% were from Asia, Latin America, and the Middle East [11]. Additionally, published data are primarily focused on newborn screening [12, 13, 14], with less research targeting the adult population. In China, regional genetic heterogeneity is evident, as variant c.2238G>C (p.W746C) predominates in southern cohorts (62.5% of alleles), while variant distribution in northern populations remains unknown [15].

This prospective, multicenter, observational cohort study aimed to identify undiagnosed LOPD within a clinically high‐risk population across Northern China by applying a standardized high‐risk profile, followed by DBS analysis for GAA enzyme deficiency and confirmatory genetic testing. Notably, this approach was intended as a pragmatic recruitment strategy that could be readily implemented by physicians with varying levels of expertise across participating hospitals to better identify LOPD and is not yet a validated prediction rule. This approach enables robust estimation of LOPD prevalence in this underrepresented region and characterization of the northern Chinese *GAA* variant spectrum.

## 2 Methods

### 2.1 Study Design and Selection of High‐Risk Criteria

This was a prospective, multicenter, observational cohort study across 39 hospitals in Northern China. Drawing on collective clinical experience in the diagnosis and management of PD, a standardized high‐risk profile for LOPD suspicion was developed [1, 7]. Selection of high‐risk criteria was based on established guidance from the Chinese Expert Consensus for diagnosis and treatment of PD [1, 7], which has been previously utilized for diagnostic criteria in retrospective studies on LOPD in China [7]. Each high‐risk criterion has corroborated screening‐value for the LOPD profile (as cited for each criteria outlined below), with the composite set of criteria aimed at more effectively capturing unidentified cases of LOPD.

Individuals meeting ≥ 2 of the following clinical features (Figure 1) were thus defined as “high‐risk” for suspected LOPD and consecutively enrolled: (a) hperCKemia (serum CK>the upper limit of normal) [16, 17], (b) limb‐girdle muscle or axial muscle weakness (Medical Research Council grade ≤ 4) [2, 18, 19, 20], (c) spinal deformity [2], (d) acute or subacute restrictive respiratory insufficiency [2, 17, 21], (e) myopathic features on electromyography (EMG) [2, 22], (f) chronic diarrhea or abdominal pain [23, 24], (g) cryptogenic stroke‐like or intracranial artery stenosis in young or middle‐aged individuals [8, 9, 25, 26, 27], (h) idiopathic pulmonary arterial hypertension (IPAH) [28, 29, 30, 31], or (i) a family history of PD [2, 17]. Criteria were handled in a binary fashion (i.e., either present or not). Though some criteria are more canonical/specific to PD (such as hyperCKemia, muscle weakness, or family history) compared to other less specific symptoms, which may be encountered more frequently in the clinical setting irrespective of PD presence (i.e., chronic diarrhea, abdominal pain), all criteria were given the same consideration. No exclusion criteria were implemented, allowing for greater real‐world generalizability.

**FIGURE 1:**
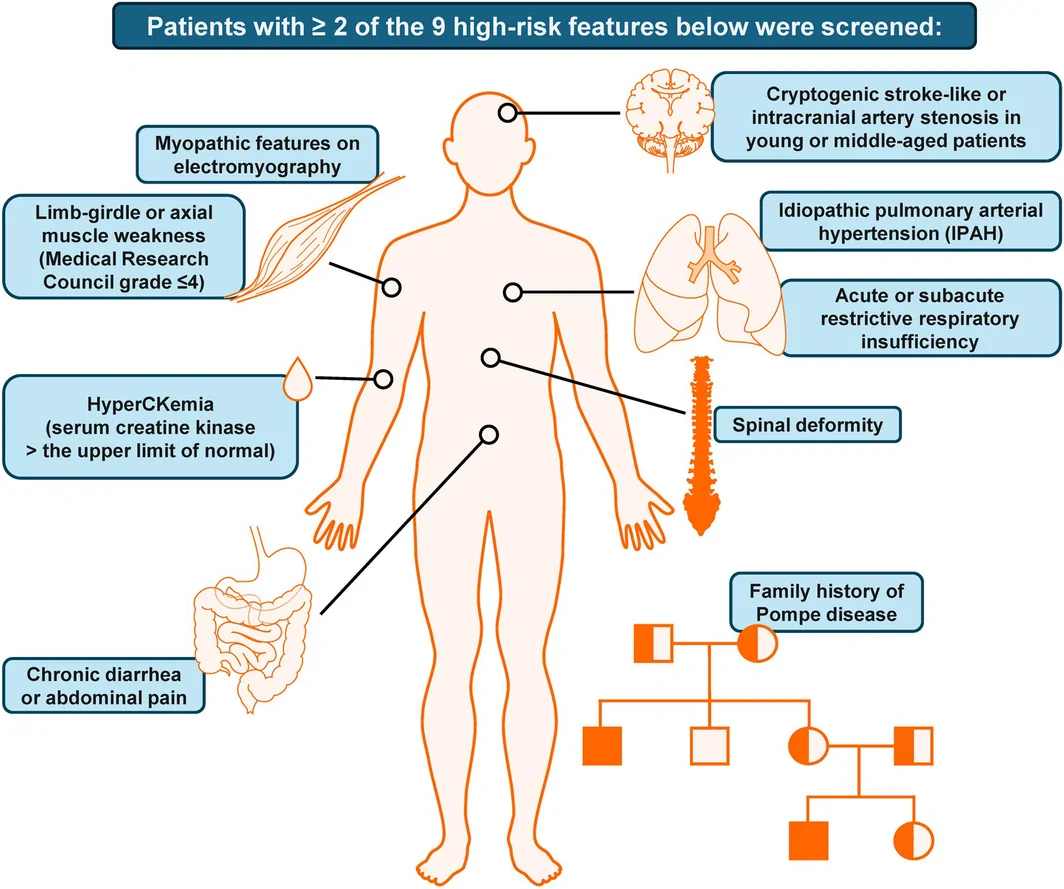
Schematic depicting the screening criteria used to identify patients at high risk for LOPD. High‐risk patients who presented with ≥ 2 of the nine features underwent DBS testing of GAA enzyme activity. If GAA enzyme deficiency was detected, LOPD diagnosis was subsequently confirmed by gene sequencing. DBS, dried blood spot; GAA, acid α‐glucosidase; LOPD, late‐onset Pompe disease.

### 2.2 GAA Enzyme Activity Assessment

Upon identification as high‐risk individuals, subjects underwent DBS sampling for GAA enzyme activity quantification. DBS analysis was performed at Suzhou Revvity Medical Laboratory (Suzhou, China) using the NeoLSD MSMS kit (Revvity, USA). Enzymatic activity was quantified via tandem mass spectrometry (MS/MS) operating in multiple reaction monitoring (MRM) mode. Target analyte signals were normalized to internal standards to determine GAA activity levels [32]. GAA enzyme deficiency was determined as < 1.46 μmol/L/h.

### 2.3 Gene Sequencing

Genomic DNA was extracted from subjects with confirmed GAA enzyme deficiency. Full‐length amplification of the *GAA* gene was performed using long‐range polymerase chain reaction. The resulting amplicons underwent next‐generation sequencing on an Illumina NovaSeq platform. Identified variants were annotated and interpreted according to American College of Medical Genetics and Genomics (ACMG)/Association for Molecular Pathology guidelines.

### 2.4 Statistical Analysis

Descriptive statistics were applied to characterize the cohort and outcomes. Continuous variables (e.g., age, enzyme activity levels) are presented as mean ± standard deviation (SD) for normally distributed data or median with interquartile range (IQR) for skewed distributions. Normality was assessed using the Shapiro–Wilk test. Categorical variables (e.g., clinical symptoms, genotype categories) were reported as frequencies and percentages. No inferential statistical comparisons were performed, consistent with the descriptive objectives of this screening study. All analyses were performed using IBM SPSS Statistics (version 26.0; IBM Corp., Armonk, NY, USA).

The nine clinical criteria (Table S1) were coded as binary variables (1 = present, 0 = absent) for each of the 46 confirmed LOPD patients; no symptom severity grading was applied. The binary matrix was uploaded to Metware Cloud (Metware Biotechnology, Wuhan, China) for heatmap visualization. To accommodate the visualization algorithm, each column (criterion) was standardized across patients (*z*‐score normalization), such that the color gradient represents the relative occurrence of each criterion across the cohort: values of 0 correspond to the cohort mean frequency of a given criterion, positive values (orange) indicate patients in whom the criterion was present more frequently than the cohort mean, and negative values (blue) indicate patients with below‐average occurrence. Rows (patients) and columns (criteria) were hierarchically clustered using Euclidean distance with Ward's minimum‐variance linkage (Ward.D2), as implemented by default in the Metware Cloud platform. Hierarchical clustering was used as an unsupervised exploratory approach to visualize patterns of criterion co‐occurrence; cluster stability was not formally tested given the descriptive nature of the analysis.

## 3 Results

### 3.1 Demographics

Based on the estimation of a high‐risk profile for LOPD, 1878 subjects were enrolled between January 2022 and December 2024 across 39 centers, spanning 12 provinces in Northern China. Of the total participants, 1044 (55.59%) were male, with a median age of 36.5 years. Overall, 46 patients (2.45%) were diagnosed with LOPD through DBS testing and confirmed by DNA sequencing, with a subset undergoing additional muscle biopsy.

### 3.2 Genetic Analysis

Genetic analysis of 46 patients with LOPD identified 55 distinct pathogenic variants across 92 alleles examined (Tables S1 and S2). The most prevalent variant was c.2238G>C (p.W746C), detected in 24 alleles (26.1%), followed by c.1309C>T (p.R437C) in 8 alleles (8.7%), and c.2662G>T (p.E888* and E888*X/Y) in 7 alleles (7.6%). Codon 746 remained a major mutational hotspot, with 30 alleles (32.6%) harboring variants at this residue, including c.2238G>C (p.W746C; 24 alleles, 26.1%), c.2237G>A (p.W746*; 2 alleles, 2.2%), c.2237G>C (p.W746S; 2 alleles, 2.2%), c.2237G>T (p.W746L; 1 allele, 1.1%), and c.2236T>G (p.W746G; 1 allele, 1.1%). Homozygosity was observed for two common variants; three patients each were homozygous for c.1309C>T (p.R437C) and c.2238G>C (p.W746C). Based on published literature and publicly available databases, six novel variants were identified in this study (c.1267_1282del [p.D423fs], c.1602dup [p.N535fs], c.‐297G>A [unknown protein], c.1546T>A [p.W516R], c.1274C>T [p.P425N], and c.752_761delinsTGCTGCCCTT [p.S251_S254delinsLLPL]).

The genotypic spectrum observed in this cohort encompassed diverse variant types. Missense variants (e.g., p.R437C, p.M439K, p.P361L, and p.W746C) predominated (69.6% of alleles, 64/92); nonsense variants (e.g., p.W746, p.Q81, p.E888*) comprised 18.5% of alleles (17/92); frameshift variants (e.g., p.E471fs, p.D423fs, p.N535fs) accounted for 3.3% (3/92); in‐frame deletions/insertions (e.g., p.M440del, p.N675del, and p.Ser251_Ser254delinsLeuLeuProLeu) represented 4.3% (4/92); and splice‐site (e.g., c.546+5G>T) and regulatory variants (e.g., c.‐32‐13T>G, c.‐297G>A) each comprised 4.3% of alleles. Recurrent variants (e.g., p.R437C, p.W746C, p.E888) and specific compound heterozygous combinations (e.g., p.Q81*/p.R437C, p.R608*/p.W746C, and p.P266S/p.W746C) were prevalent. Based on ACMG variant classification per previous reports, the vast majority of variants were deemed pathogenic/likely pathogenic, with 10 variants of uncertain significance (Table S2).

### 3.3 Clinical and Laboratory Characteristics

Of the 46 patients, 60.9% were female (*n* = 28) and 39.1% were male (*n* = 18). Neuromuscular manifestations were the predominant clinical feature, with limb‐girdle or axial muscle weakness present in 89.1% of patients (*n* = 41), whereas hyperCKemia was observed in 73.9% (*n* = 34). Respiratory compromise was a significant feature, with acute or subacute restrictive respiratory insufficiency observed in 50% of patients (*n* = 23). EMG revealed myogenic lesions in 23.9% of patients (*n* = 11) and spinal deformity in 17.4% of patients (*n* = 8). Systemic involvement included chronic diarrhea and/or abdominal pain in 8.7% of patients (*n* = 4), as well as rare vascular events, with cryptogenic stroke‐like or intracranial stenosis and IPAH reported in 4.3% (*n* = 2) of patients each. A family history of PD was noted in 6.5% of patients (*n* = 3). Biochemical analysis demonstrated total elevated serum CK (mean 856.3 U/L ± 929.6) and deficient GAA activity (mean 0.36 μmol/L/h ± 0.18), consistent with LOPD pathophysiology.

Heatmap analysis (Figure 2) revealed distinct patterns in the prevalence and co‐occurrence of nine key clinical features among the 46 patients with suspected or confirmed LOPD. The most prevalent manifestation was limb‐girdle muscle or axial muscle weakness (b), observed in over 80% of patients. HyperCKemia (a) was also highly common, co‐occurring with muscle weakness in nearly 63% of cases. Spinal deformity (c) was noted as the reason 17.4% of patients were enrolled and was consistently associated with limb‐girdle muscle or axial muscle weakness phenotypes (b). Acute or subacute restrictive respiratory involvement (d), reflecting respiratory muscle insufficiency, was evident in almost 50% of cases and frequently co‐occurred with limb‐girdle muscle or axial muscle weakness (b). Chronic diarrhea or abdominal pain (f) was observed in 8.7% of cases, often alongside multisystemic involvement. In contrast, cryptogenic stroke‐like or intracranial artery stenosis (g) and IPAH (h), symptoms not typically recognized as indicators of LOPD by most clinicians, were rare, occurring in fewer than 10% of cases.

**FIGURE 2:**
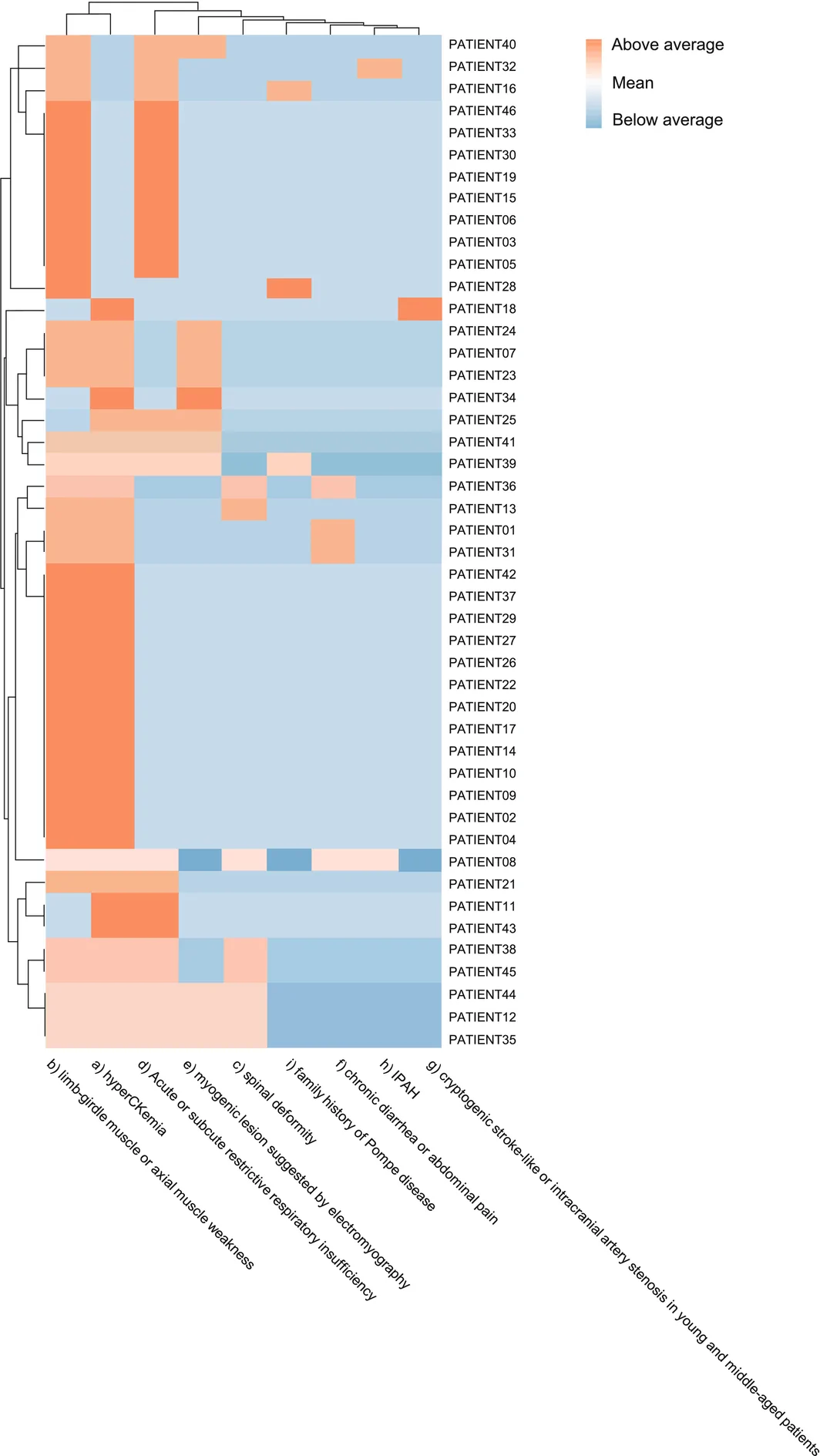
Symptom profile clustering of patients. Heatmap generated using Metware Cloud. The nine clinical high‐risk criteria were recorded as binary variables (present/absent); for visualization, each criterion was *z*‐score standardized across patients, and the color gradient indicates the relative occurrence of each criterion (orange = above cohort mean, blue = below cohort mean). Rows (patients) and columns (criteria) were hierarchically clustered (Euclidean distance, Ward's minimum‐variance linkage). IPAH, idiopathic pulmonary arterial hypertension.

## 4 Discussion

This study represents the largest investigation of LOPD screening conducted in China to date, with 1878 subjects enrolled. The data collected over the 3‐year screening period provide valuable epidemiological insights into the prevalence of LOPD within the region. Furthermore, the findings reveal critical aspects of the current diagnostic and treatment landscape for PD in Northern China. Collectively, these results highlight the ongoing challenges in attaining a timely diagnosis and the urgent need for improved management strategies for this rare metabolic disorder.

### 4.1 Distinct Genetic Architecture and Implications

Consistent with other reports of Chinese patients, c.2238G>C (p.W746C) was the most prominent variant reported in this cohort (26.1% allele frequency) [7, 33, 34]. Notably, codon 746 constitutes a significant mutational hotspot, accounting for 33% of pathogenic variants in this cohort through diverse variants, including p.W746C, p.W746*, p.W746S, p.W746L, and p.W746G. This clustering suggests unique population‐specific evolutionary pressures or founder effects within Chinese patients.

These findings stand in contrast to the prevalence of the c.‐32‐13T>G splice‐site variant, which is present in approximately 70% of Caucasian patients with LOPD [1, 10, 35]. The c.‐32‐13T>G variant, which is considered relatively “mild” and presents with less respiratory involvement [10], was only present in one heterozygous patient in this cohort (1.1% allele frequency). Interestingly, this patient did not present with respiratory insufficiency. Furthermore, in two studies involving 19 patients with LOPD from Southern China, variant c.1935C>A p.(Asp645Glu) was identified as the second most common pathogenic variant [34, 36], but it was not detected in this Northern Chinese cohort. These findings underscore the importance of developing regionally tailored screening panels that prioritize high‐frequency pathogenic variants (e.g., codon 746 variants), which could, in turn, substantially improve diagnostic efficiency in resource‐constrained settings.

The identification of homozygous cases (three patients each for p.W746C and p.R437C) provided insights into potential genotype–phenotype correlations. Of the three patients who were homozygous for c.2238G>C (p.W746C), two exhibited hyperCKemia, all displayed limb‐girdle muscle or axial muscle weakness, two had acute or subacute restrictive respiratory insufficiency, and one presented with a myogenic lesion indicated by EMG (Table S1). Among the three patients who were homozygous for variant c.1309C>T (p.R437C), all presented with limb‐girdle muscle or axial muscle weakness and acute or subacute restrictive respiratory insufficiency. Further genotype–phenotype correlation studies are necessary to validate these observations. A systematic genotype–phenotype analysis was not performed here, owing to the small number of patients within each variant subgroup and the heterogeneity of assessments across centers. This will be addressed in future studies with standardized protocols and an expanded cohort.

### 4.2 Clinical Applicability of the Multisystem Screening Paradigm

It is important to acknowledge that the high‐risk criteria used in this study to identify patients represent a pragmatic recruitment strategy rather than a validated clinical prediction rule. Nonetheless, the inclusion of non‐classical indicators, specifically cryptogenic stroke‐like or intracranial stenosis (g), IPAH (h), and chronic diarrhea or abdominal pain (f), proved invaluable in identifying atypical LOPD presentations. Although individually rare (each found in ≤ 4.3% of individuals here), these manifestations collectively facilitated 17.4% of diagnoses, underscoring the clinical significance of these phenotypes. The observed gastrointestinal symptoms (8.7%) align with emerging evidence of enteric nervous system involvement in PD [24], while the occurrence of cerebrovascular events in young adults broadens the recognized phenotypic spectrum beyond classical myopathic manifestations [37]. Although limited, existing literature corroborates this report of intracranial vasculopathy and pulmonary hypertension in patients with LOPD [9, 28, 37]. Importantly, spinal deformities (c) were observed in 17.4% of patients and consistently occurred alongside limb‐girdle muscle weakness. This pattern reinforces their role as secondary markers of progressive myopathy rather than isolated features. Together, these observations strongly support the inclusion of spinal deformities in future screening protocols to help identify diagnostically challenging cases.

### 4.3 Diagnostic Advancements and Clinical Implementation

The efficacy of targeted screening is evidenced by the higher observed prevalence of LOPD (2.45%) within this cohort than the estimated incidence in China's general population (1:40 000) [5]. Previous studies report diagnostic delays exceeding 7 years in China [7], primarily attributable to nonspecific presentations (e.g., isolated hyperCKemia or respiratory insufficiency). This protocol facilitates earlier detection by triggering diagnostic evaluation in individuals fulfilling ≥ 2 criteria, even without classic limb‐girdle muscle weakness. For example, individuals presenting with cryptogenic stroke plus hyperCKemia (criteria a + g) or IPAH with myopathic EMG findings (e + h) could achieve diagnosis 5–8 years earlier than through conventional pathways [33]. Implementation of this standardized profile across neurology, pulmonology, and gastroenterology practices could improve diagnostic odysseys and clinical trajectories.

### 4.4 Limitations and Future Research Directions

While this study provides robust regional epidemiological data, several methodological considerations warrant acknowledgment. The recruitment primarily from tertiary referral centers may overrepresent severe phenotypes, potentially inflating prevalence estimates. Longitudinal follow‐up is imperative to establish genotype–phenotype correlations, particularly for the prevalent p.W746C variant, and to evaluate differential responses to ERT in atypically presenting subgroups (e.g., stroke or IPAH patients). Technical limitations in detecting non‐coding variants, copy number variations, and structural rearrangements [4] may underlie undiagnosed cases with suggestive clinical features. Future investigations should prioritize long‐read whole genome sequencing to resolve complex allelic architectures and include multi‐omics integration (e.g., transcriptomics for splicing defect characterization) [5] to refine diagnostic precision.

Statistical comparisons were limited in this study design. First and foremost, the analysis lacked a non‐LOPD comparator group. Further, future investigations should include receiver operating characteristic (ROC) analyses, as well as evaluation of odds ratios with 95% confidence intervals for individual criterion and for clinically relevant combinations such as hyperCKemia plus limb‐girdle/axial weakness and muscle weakness plus restrictive respiratory insufficiency. Likewise, as individuals fulfilling fewer than two screening criteria were not enrolled, validation of the sensitivity for the ≥ 2‐criterion threshold may be limited. Lastly, cost‐effectiveness analyses could facilitate scalable implementation across China's heterogeneous healthcare systems. Such advances would improve early diagnosis and personalized management strategies for LOPD.

## Author Contributions

Yuying Zhao was involved in planning, conducting, and reporting the study. All authors contributed to the article and approved the submitted version.

## Funding

This publication was sponsored by Sanofi through a Sanofi Medical Award to help with manuscript preparation and publication. Writing support was provided by Devorah Natelson, PhD, of IMPRINT Science, New York, NY, USA, and was funded by Sanofi.

## Ethics Statement

This study was reviewed and approved by the Ethical Advisory Committee of Qilu Hospital. Shandong University approved this study (KYLL‐202207‐027‐1).

## Consent

Informed written consent was obtained from each individual or their legally authorized representative.

## Conflicts of Interest

The authors declare that the research was conducted in the absence of any commercial or financial relationships that could be construed as a potential conflicts of interest. The research conducted was not funded through Sanofi. The authors disclose that Sanofi provided funding through a medical award for manuscript preparation and publication, for medical writing support only. Sanofi had no role in data collection, data analysis, or interpretation of the data.

## Supporting information


**Table S1:** Genetic and phenotypic characteristics of patients with LOPD.
**Table S2:** Detected variant classification. Shaded rows represent novel variants.

## Data Availability

The data that support the findings of this study are available in the Supplementary Material of this article.
