# Supplementary material for: High‐Risk Screening for Late‐Onset Pompe Disease in Northern China: Prevalence, Genotypic Spectrum, and Diagnostic Challenges
Source: JIMD Rep. 2026 Nov 13;67(6):e70138. doi: 10.1002/jmd2.70138 (PMC13768598; doi:10.1002/jmd2.70138)
Supplement: Supplementary file 1 [file JMD2-67-e70138-s001.docx]

**Supplementary Table S1.** Genetic and phenotypic characteristics of patients with LOPD.

| Variant 1 | Variant 2 | Number of patients | Family history of PD | Average GAA enzyme activity (μmol/L/h） | Average CK  (U/L) | HyperCKemia  (serum CK > the upper limit of normal) | Limb-girdle muscle or axial muscle weakness  (Medical Research Council grade ≤4) | Spinal deformity | Acute or subacute restrictive respiratory insufficiency | Myopathic features on EMG | Chronic diarrhea or abdominal pain | Cryptogenic stroke-like or intracranial artery stenosis in young and middle-aged patients | Idiopathic pulmonary arterial hypertension |
| --- | --- | --- | --- | --- | --- | --- | --- | --- | --- | --- | --- | --- | --- |
| c.2238G>C  (p.W746C) | c.2238G>C  (p.W746C) | 3 | x | 0.55 | 599 | (2/3) | (3/3) | x | (2/3) | (1/3) | x | x | x |
| c.2238G>C  (p.W746C) | c.2662G>T  (p.E888*/Y/X) | 3 | x | 0.36 | 754 | (3/3) | (2/3) | x | x | x | x | x | x |
| c.1309C>T  (p.R437C) | c.1309C>T  (p.R437C) | 3 | (1/3) | 0.38 | 270 | x | (3/3) | x | (3/3) | x | x | x | x |
| c.2238G>C  (p.W746C) | c.1316T>A  (p.M439K) | 2 | x | 0.41 | 590 | (2/2) | (1/2) | (1/2) | (1/2) | x | x | x | x |
| c.2238G>C  (p.W746C) | c.1121G>A  (p.C374Y) | 1 | x | 0.21 | 2,421 | (1/1) | x | x | (1/1) | (1/1) | x | x | x |
| c.2238G>C  (p.W746C) | c.1082C>T  (p.P361L) | 1 | x | 0.18 | 356 | (1/1) | (1/1) | x | x | x | x | x | x |
| c.2238G>C  (p.W746C) | c.241C>T  (p.Q81*) | 1 | x | 0.76 | 3,969 | (1/1) | (1/1) | x | x | x | x | x | x |
| c.2238G>C  (p.W746C) | c.1411_1414delGAGA  (p.E471fs) | 1 | x | 0.67 | 369 | x | (1/1) | x | (1/1) | x | x | x | x |
| c.2238G>C  (p.W746C) | c.1822C>T  (p.R608*) | 1 | x | 0.26 | 1,650 | (1/1) | (1/1) | x | x | (1/1) | x | x | x |
| c.2238G>C  (p.W746C) | c.1267_1282del  (p.D423fs) | 1 | x | 0.35 | 391 | (1/1) | (1/1) | (1/1) | (1/1) | x | x | x | x |
| c.2238G>C  (p.W746C) | c.1602dup  (p.N535fs) | 1 | x | 0.29 | 96 | x | (1/1) | x | (1/1) | x | x | x | x |
| c.2238G>C  (p.W746C) | c.2237G>C  (p.W746S) | 1 | x | 0.25 | 512 | (1/1) | (1/1) | x | x | x | x | x | x |
| c.2238G>C  (p.W746C) | c.2237G>T  (p.W746L) | 1 | x | 0.6 | 552 | x | (1/1) | x | (1/1) | x | x | x | (1/1) |
| c.2238G>C  (p.W746C) | c.796C>T  (p.P266S) | 1 | x | 0.34 | 214 | x | (1/1) | x | (1/1) | x | x | x | x |
| c.2238G>C  (p.W746C) | c.1933G>A (p.D645N) | 1 | x | 0.41 | 842 | (1/1) | (1/1) | (1/1) | (1/1) | (1/1) | x | x | x |
| c.2238G>C  (p.W746C) | c.1843G>A (p.G615R) | 1 | x | 0.26 | 1,160 | (1/1) | x | x | x | (1/1) | x | x | x |
| c.2238G>C  (p.W746C) | c.2237G>A (p.W746*) | 1 | x | 0.27 | 429 | (1/1) | (1/1) | x | x | x | x | x | x |
| c.1309C>T  (p.R437C) | c.241C>T  (p.Q81*) | 1 | x | 0.92 | 279 | x | (1/1) | x | (1/1) | x | x | x | x |
| c.1309C>T  (p.R437C) | c.2662G>T (p.E888*) | 1 | x | 0.22 | 558 | (1/1) | x | x | x | x | x | (1/1) | x |
| c.1082C>T  (p.P361L) | c.796C>T (p.P266S) | 1 | x | - | 1,292 | (1/1) | (1/1) | (1/1) | (1/1) | (1/1) | x | x | x |
| c.1100G>A  (p.W367*) | c.-297G>A  (p.?) | 1 | x | 0.47 | 1,383 | (1/1) | (1/1) | x | x | x | (1/1) | x | x |
| c.1153C>T  (p.R385C) | c.1546T>A (p.W516R) | 1 | x | 0.2 | 796 | (1/1) | (1/1) | (1/1) | x | x | (1/1) | x | x |
| c.1316T>A (p.M439K) | c.2662G>T (p.E888*) | 1 | x | 0.17 | 361 | (1/1) | (1/1) | x | x | (1/1) | x | x | x |
| c.1320_1322del (p.M440del) | c.1622C>T (p.P541L) | 1 | x | 0.35 | 282 | (1/1) | (1/1) | x | x | x | x | x | x |
| c.1465G>A (p.D489N) | c.1274C>T  (p.P425N) | 1 | x | 0.1 | 889 | (1/1) | x | x | (1/1) | x | x | x | x |
| c.1585T>C (p.S529P) | c.1837T>C (p.W613R) | 1 | x | 0.51 | 728 | (1/1) | (1/1) | x | x | x | x | x | x |
| c.1735G>A (p.E579K) | c.1219T>C (p.Y407H) | 1 | x | 0.42 | 873 | (1/1) | (1/1) | x | (1/1) | (1/1) | x | x | x |
| c.1780C>T (p.R594C) | c.1799G>C (p.R600P) | 1 | x | 0.19 | 520 | (1/1) | (1/1) | (1/1) | (1/1) | (1/1) | x | x | x |
| c.1814G>A (p.G605D) | c.2662G>T (p.E888*) | 1 | (1/1) | 0.23 | 1,167 | (1/1) | (1/1) | x | (1/1) | (1/1) | x | x | x |
| c.1822C>T (p.R608*) | c.752_761delinsTGCTGCCCTT (p.S251_S254  delinsLLPL) | 1 | x | 0.23 | 94 | x | (1/1) | x | (1/1) | x | x | x | x |
| c.1871_1872delTCinsAG (p.L624Q) | c.2237G>A (p.W746*) | 1 | x | 0.37 | 662 | (1/1) | (1/1) | x | x | x | (1/1) | x | x |
| c.2024_2026del (p.N675del) | c.2051C>A (p.P684Q) | 1 | x | 0.54 | 762 | x | (1/1) | x | (1/1) | x | x | x | x |
| c.2237G>C (p.W746S) | c.2105G>A (p.R702H) | 1 | x | 0.15 | 510 | (1/1) | (1/1) | (1/1) | (1/1) | x | x | x | x |
| c.2240G>A* (p.G747Q) | c.1822C>T (p.R608*) | 1 | x | 0.31 | 387 | (1/1) | (1/1) | x | x | x | x | x | x |
| c.241C>T  (p.Q81*) | c.1925T>A (p.V642D) | 1 | x | 0.25 | 611 | (1/1) | (1/1) | x | x | (1/1) | x | x | x |
| c.361C>T  (p.Q121*) | c.2236T>G (p.W746G) | 1 | (1/1) | 0.34 | 67 | x | (1/1) | x | x | x | x | x | x |
| c.503G>C  (p.R168P) | c.-32-13T>G  (p.?) | 1 | x | 0.32 | 352 | (1/1) | (1/1) | x | x | x | x | x | x |
| c.546+5G>T  (p.?) | c.2662G>T (p.E888*) | 1 | x | 0.3 | 4,500 | (1/1) | (1/1) | x | x | x | x | x | x |
| c.546G>T  (p.T182=) | c.2024_2026del (p.N675del) | 1 | x | 0.25 | 2,808 | (1/1) | (1/1) | x | x | x | x | x | x |

“x” denotes absence of phenotype. “-” denotes data not available. Note: novel variants reported in this study denoted by shading.

*Previously reported in Li et al 2026 as p.G747E.

CK, creatine kinase; EMG, electromyography; GAA, acid α-glucosidase; LOPD, late-onset Pompe disease; PD, Pompe disease.

**Supplementary Table S2.** Detected variant classification. Shaded rows represent novel variants.

| **Variant** | **Allele Frequency (n, %)** | **ACMG Classification** | **ClinVar Database Identifier / Variation ID** |
| --- | --- | --- | --- |
| c.2238G>C  (p.W746C) | 24 (26.1%) | Pathogenic | NM_000152.5(GAA):c.2238G>C (p.Trp746Cys) / Variation ID: 265160 |
| c.2662G>T  (p.E888*/Y/X) | 3 (3.3%) | Pathogenic | NM_000152.5(GAA):c.2662G>T (p.Glu888Ter) / Variation ID: 578595 |
| c.1309C>T  (p.R437C) | 8 (8.7%) | Pathogenic | NM_000152.5(GAA):c.1309C>T (p.Arg437Cys) / Variation ID: 189082 |
| c.1316T>A  (p.M439K) | 3 (3.3%) | Pathogenic | NM_000152.5(GAA):c.1316T>A (p.Met439Lys) / Variation ID: 371305 |
| c.1121G>A  (p.C374Y) ^†^ | 1 (1.1%) | Likely pathogenic | [reported in Reuser et al, 2019](Reuser et al 2019) |
| c.1082C>T  (p.P361L) | 2 (2.2%) | Pathogenic | NM_000152.5(GAA):c.1082C>T (p.Pro361Leu) / Variation ID: 403712 |
| c.241C>T  (p.Q81*) | 3 (3.3%) | Pathogenic | NM_000152.5(GAA):c.241C>T (p.Gln81Ter) / Variation ID: 557360 |
| c.1411_1414delGAGA  (p.E471fs) | 1 (1.1%) | Pathogenic | NM_000152.5(GAA):c.1411_1414del (p.Glu471fs) / Variation ID: 188874 |
| c.1822C>T  (p.R608*) | 3 (3.3%) | Pathogenic | NM_000152.5(GAA):c.1822C>T (p.Arg608Ter) / Variation ID: 972803 |
| c.1267_1282del  (p.D423fs) | 1 (1.1%) | - | - |
| c.1602dup  (p.N535fs) | 1 (1.1%) | - | - |
| c.2237G>C  (p.W746S) | 2 (2.2%) | Likely pathogenic | NM_000152.5(GAA):c.2237G>C (p.Trp746Ser)/ Variation ID: 188484 |
| c.2237G>T  (p.W746L) | 1 (1.1%) | Uncertain significance | NM_000152.5(GAA):c.2237G>T (p.Trp746Leu) / Variation ID: 284776 |
| c.796C>T  (p.P266S) | 2 (2.2%) | Likely pathogenic | NM_000152.5(GAA):c.796C>T (p.Pro266Ser) / Variation ID: 556117 |
| c.1933G>A  (p.D645N) | 1 (1.1%) | Pathogenic | NM_000152.5(GAA):c.1933G>A (p.Asp645Asn) / Variation ID: 188728 |
| c.1843G>A  (p.G615R) | 1 (1.1%) | Pathogenic | NM_000152.5(GAA):c.1843G>A (p.Gly615Arg) / Variation ID: 188786 |
| c.2237G>A  (p.W746*) | 2 (2.2%) | Pathogenic | NM_000152.5(GAA):c.2237G>A (p.Trp746Ter) / Variation ID: 280063 |
| c.2662G>T  (p.E888*) | 4 (4.3%) | Pathogenic | NM_000152.5(GAA):c.2662G>T (p.Glu888Ter) / Variation ID: 578595 |
| c.1100G>A  (p.W367*) | 1 (1.1%) | Pathogenic | NM_000152.5(GAA):c.1100G>A (p.Trp367Ter) / Variation ID: 972773 |
| c.-297G>A  (p.?) | 1 (1.1%) | - | - |
| c.1153C>T  (p.R385C) | 1 (1.1%) | Uncertain significance | NM_000152.5(GAA):c.1153C>T (p.Arg385Cys) / Variation ID: 575359 |
| c.1546T>A  (p.W516R) | 1 (1.1%) | - | - |
| c.1320_1322del (p.M440del) | 1 (1.1%) | Likely pathogenic | NM_000152.5(GAA):c.1317GAT[1] (p.Met440del) / Variation ID: 526518 |
| c.1622C>T  (p.P541L)^†^ | 1 (1.1%) | - | [Reported in: Li et al, 2026](Li et al 2026) |
| c.1465G>A  (p.D489N) | 1 (1.1%) | Pathogenic | NM_000152.5(GAA):c.1465G>A (p.Asp489Asn) / Variation ID: 92465 |
| c.1274C>T  (p.P425N) | 1 (1.1%) | - | - |
| c.1585T>C  (p.S529P) | 1 (1.1%) | Pathogenic | NM_000152.5(GAA):c.1585T>C (p.Ser529Pro) / Variation ID: 645572 |
| c.1837T>C  (p.W613R) | 1 (1.1%) | Uncertain significance | NM_000152.5(GAA):c.1837T>C (p.Trp613Arg) / Variation ID: 2821438 |
| c.1735G>A  (p.E579K) | 1 (1.1%) | Pathogenic | NM_000152.5(GAA):c.1735G>A (p.Glu579Lys) / Variation ID: 495664 |
| c.1219T>C  (p.Y407H) | 1 (1.1%) | Uncertain significance | NM_000152.5(GAA):c.1219T>C (p.Tyr407His) / Variation ID: 167112 |
| c.1780C>T  (p.R594C) | 1 (1.1%) | Uncertain significance | NM_000152.5(GAA):c.1780C>T (p.Arg594Cys) / Variation ID: 662598 |
| c.1799G>C  (p.R600P) | 1 (1.1%) | Likely pathogenic | NM_000152.5(GAA):c.1799G>C (p.Arg600Pro) / Variation ID: 4817584 |
| c.1814G>A  (p.G605D) ^†^ | 1 (1.1%) | Uncertain significance | [Reported in Muraoka et al, 2011](Muraoka et al 2011) |
| c.752_761delinsTGCTGCCCTT (p.S251_S254  delinsLLPL) | 1 (1.1%) | Likely pathogenic | - |
| c.1871_1872delTCinsAG (p.L624Q) ^†^ | 1 (1.1%) | - | [Reported in: Li et al, 2026](Li et al 2026) |
| c.2024_2026del (p.N675del) | 2 (2.2%) | Pathogenic | NM_000152.5(GAA):c.2021ACA[1] (p.Asn675del) / Variation ID: 189006 |
| c.2051C>A  (p.P684Q) | 1 (1.1%) | Uncertain significance | NM_000152.5(GAA):c.2051C>A (p.Pro684Gln) / Variation ID: 1683224 |
| c.2105G>A  (p.R702H) | 1 (1.1%) | Pathogenic | NM_000152.5(GAA):c.2105G>A (p.Arg702His) / Variation ID: 426278 |
| c.2240G>A  (p.G747Q) ^†^ | 1 (1.1%) | Likely pathogenic | [Reported in: Li et al, 2026](Li et al 2026) |
| c.1925T>A  (p.V642D) | 1 (1.1%) | Uncertain significance | NM_014844.5(TECPR2):c.1925T>A (p.Val642Asp) / Variation ID: 989674 |
| c.361C>T  (p.Q121*) | 1 (1.1%) | Likely pathogenic | NM_000152.5(GAA):c.361C>T (p.Gln121Ter) / Variation ID: 1369778 |
| c.2236T>G  (p.W746G) | 1 (1.1%) | Pathogenic | NM_000152.5(GAA):c.2236T>G (p.Trp746Gly) / Variation ID: 556431 |
| c.503G>C  (p.R168P) | 1 (1.1%) | Uncertain significance | NM_000152.5(GAA):c.503G>C (p.Arg168Pro) / Variation ID: 639915 |
| c.-32-13T>G  (p.?) | 1 (1.1%) | Pathogenic | NM_000152.5(GAA):c.-32-13T>G / Variation ID: 4027 |
| c.546+5G>T  (p.?) | 1 (1.1%) | Uncertain significance | NM_000152.5(GAA):c.546+5G>T / Variation ID: 557811 |
| c.546G>T  (p.T182=) | 1 (1.1%) | Pathogenic | NM_000152.5(GAA):c.546G>T (p.Thr182=) / Variation ID: 370637 |

^†^Does not appear in ClinVar database but has been reported previously as cited.

Note: novel variants reported in this study denoted by shading.

ACMG, American College of Medical Genetics and Genomics.
